# Cyst of the Canal of Nuck: a case report

**DOI:** 10.1093/jscr/rjae374

**Published:** 2024-05-31

**Authors:** Tsonka Lukanova, Ivelin Takorov, Elina Todorova, Ani Dzakova

**Affiliations:** First Clinic of Abdominal Surgery, Military Medical Academy, Sofia 1606, Bulgaria; First Clinic of Abdominal Surgery, Military Medical Academy, Sofia 1606, Bulgaria; First Clinic of Abdominal Surgery, Military Medical Academy, Sofia 1606, Bulgaria; Clinic of Plastic Surgery and Burns, Military Medical Academy, Sofia 1606, Bulgaria

**Keywords:** Canal of Nuck, cyst, surgical management

## Abstract

The cyst of the Canal of Nuck, or hydrocele, is a rare pathological condition in the female inguinal region. We present a 44-year-old female with a cystic lesion in the right inguinal area, detailing clinical symptoms, differential diagnosis and imaging findings. Surgical intervention involved complete cyst excision, with no recurrence during the follow-up. This case underscores the importance of accurate diagnosis and targeted surgical treatment for favourable outcomes in managing rare anatomical variations like the Canal of Nuck cyst.

## Introduction

The Canal of Nuck cyst represents an infrequent entity for mass observed in the female inguinal region. Given the uncommon nature of the Canal of Nuck cyst, combined with the possibility of underdiagnosis and its prevalence in pediatric populations, there is no statistical data on the occurrence among adults [1]. Regarding this, there is no standardised surgical procedure to be performed. Most cases of the condition are successfully cured with complete excision with no relapse.

## Case presentation

**Figure 1:**
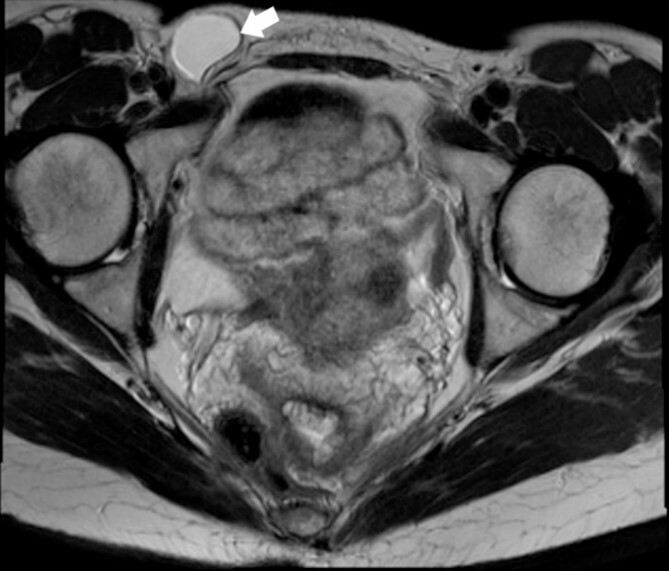
MRI of the Cyst of the Canal of Nuck–T1 sequence. This image highlights the cyst (indicated by the white arrow) within the inguinal region.

**Figure 2:**
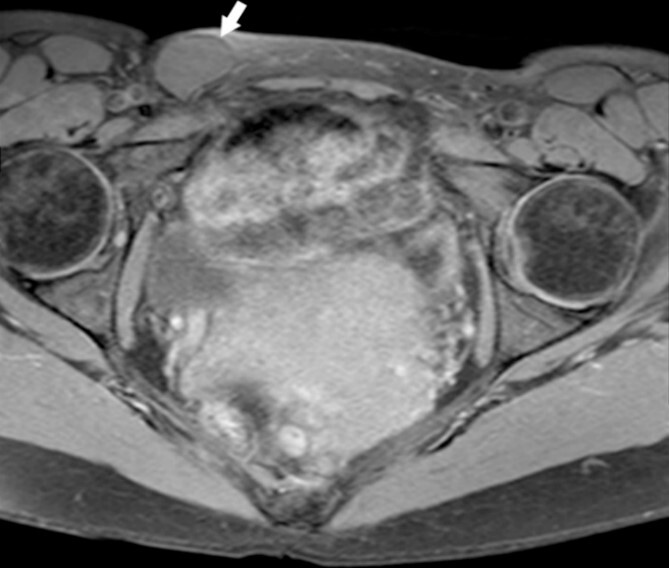
MRI of the Cyst of the Canal of Nuck–T2 sequence. This figure displays the cyst (indicated by the white arrow), showcasing its appearance on a T2-weighted image.

A 44-year-old Caucasian woman presented in our clinic with a painless swelling in the right inguinal region that appeared 10 months before her hospitalisation. A small soft mass measuring 4 × 2 cm^2^ was found on the physical examination. There were no changes in size or shape in the lesion when performing the Valsalva manoeuvre. Magnetic resonance imaging (MRI) showed an oval cyst measured 3.9/2.6/2.4 cm close to the uterus’s round ligament in the right inguinal channel. The lesion was hypointense in T1- (Fig. 1) and hyperintense in T2-sequence (Fig. 2). An anechoic cyst was observed using ultrasound. The cyst was classified as type I based on Counseller and Black’s classification [2]. Blood analysis showed no pathological changes.

**Figure 3:**
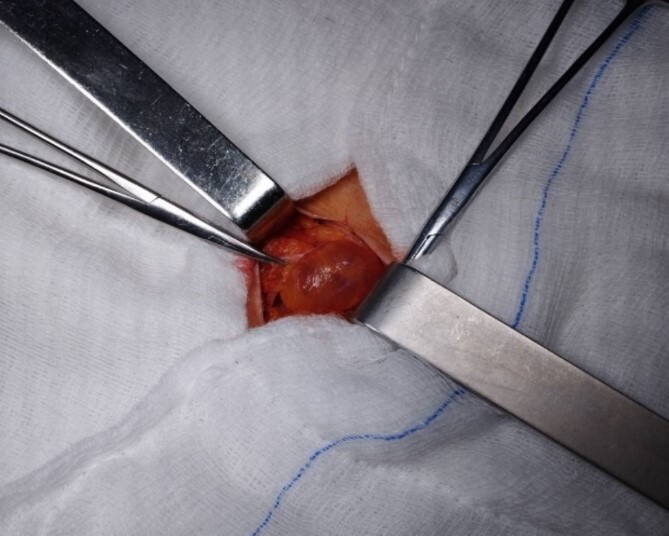
Intraoperative view of the cyst of the Canal of Nuck. This image directly represents the cyst during surgical excision and offers valuable insights into its size and location.

With a small right para-inguinal incision, a small sample cyst along the round ligament was identified with no communication with the peritoneal cavity. It was dissected from the ligament and completely excised (Fig. 3). The inguinal canal was closed by suturing the tendon of the external abdominal oblique to the edge of the inguinal ligament. The pathological examination revealed a wall of a benign cyst, with focally inflamed mesothelial lining and mononuclear inflammation, and confirmed the diagnosis of a cyst of the Canal of Nuck. The postoperative period was uneventful. The patient was discharged the day after the surgical procedure. Twelve months later, there is no evidence of relapse.

## Discussion

The ‘cyst of the canal of Nuck’ was first documented in 1691 by the Dutch anatomist Anton Nuck van Leiden. He described it as a female entity analogical to the male’s patent vaginal process of the inguinal canal [3]. If the obliteration of the vaginal process that accompanies the round ligament through the inguinal canal into the labium majus deteriorates, a hydrocele due to accumulation of peritoneal fluid occurs, leading to a ‘hydrocele’ or ‘cyst’ of the canal of Nuck (both are synonyms) [4].

In evaluating a palpable mass in the inguinal region, the differential diagnosis encompasses a variety of conditions, each with distinct diagnostic markers and clinical presentations. This analysis aims to provide a comprehensive comparison, emphasising features beyond the changes observed during menstruation or the Valsalva manoeuvre, to guide clinicians toward a more accurate diagnosis [5].

### Inguinal hernia

Characterized by a cough impulse, increasing in size with standing or abdominal straining. Hernias often have a direct association with the inguinal canal and may retract when lying down.

### Lipoma or leiomyoma

Benign soft tissue tumours that are soft, mobile and non-tender. They are easily distinguished from cysts of the canal of Nuck due to their lack of relation to the menstrual cycle or Valsalva manoeuvre.

### Ganglion cyst

Rare in the inguinal region; these are smooth, firm and possibly trans-illuminated, unlike the Canal of Nuck cysts.

### Lymphadenopathy

Enlarged lymph nodes may indicate infection or malignancy, distinguished from a Canal of Nuck cyst by firmness and potential systemic symptoms.

### Bartholin’s cyst

Tenderness and infection association contrasts with the typically non-tender, non-infectious Canal of Nuck cyst.

### Endometriosis of the round ligament

Presents with cyclical pain correlating with menstrual periods, not seen in the Canal of Nuck cysts.

### Cyst of the Canal of Nuck

This cyst is distinguished by its location along the path of the embryonic Canal of Nuck and its lack of significant change in size during menstruation or the Valsalva manoeuvre. The absence of skin infection signs in the inguinal region supports this diagnosis. Ultrasound imaging is pivotal, showing a fluid-filled sac, which MRI can confirm to delineate its relationship with surrounding tissues. The cyst of the Canal of Nuck is a thin-walled structure with hypointense T1-signal and hyperintense T2-signal characteristics [5, 6].

Three types of hydroceles—encysted, communicating and bilocular—were classified by Counseller and Black in 1941 [2]. The most common type, the encysted, is presented by a confined round cyst that does not have communication with the peritoneal cavity—type I. followed by a ‘coma’-shaped cyst, whose ‘tail-like’ part extends into the peritoneum—type II. The last variation is type III—an ‘hourglass’ formation—when the deep inguinal ring constricts and separates the cyst into two parts—one that communicates with the peritoneal cavity and one that does not.

The gold standard of treatment of cysts of the Canal of Nuck is complete excision of the mass. In the medical literature, most of these patients have been treated with open surgery, but several laparoscopic cases have been reported [7]. Choosing between open and laparoscopic techniques for the excision of a Canal of Nuck cyst significantly impacts surgical outcomes and postoperative management. Laparoscopic surgery, favoured for its minimally invasive nature, leads to smaller incisions, reduced postoperative discomfort and quicker recovery, thus facilitating an earlier return to daily activities. However, this approach demands specialised surgical skills and equipment, with potential risks including port site infections or accidental injury to nearby structures. Conversely, open surgery, despite its longer recovery period and higher risks of wound-related complications, allows for precise cyst removal through direct visualisation.

It is essential to state that the cyst of the Canal of Nuck is confirmed after pathological evaluation. Notably, the cyst of the Canal of Nuck in our presented case showed no recurrence, similar to findings reported in the broader literature [7, 8], highlighting the efficacy of the surgical approach used. This direct correlation underscores the relevance of our case study within the context of existing medical discussions and supports the continued examination of surgical outcomes for such rare conditions.

## Conclusion

Once regarded as a rarity, the cyst of the Canal of Nuck is increasingly diagnosed, reflecting advancements in diagnostic imaging and the enhanced skills of medical practitioners. Precise diagnosis is crucial for effective surgical management. Complete removal of the cyst consistently results in favourable outcomes. This case exemplifies the importance of thorough diagnostic assessment and tailored surgical approaches in managing rare conditions, offering valuable insights into both procedural efficacy and patient care strategies.
